# Analysis of smooth pursuit eye movements in a clinical context by tracking the target and eyes

**DOI:** 10.1038/s41598-022-12630-6

**Published:** 2022-05-19

**Authors:** Masakazu Hirota, Kanako Kato, Megumi Fukushima, Yuka Ikeda, Takao Hayashi, Atsushi Mizota

**Affiliations:** 1 Department of Orthoptics, Faculty of Medical Technology, Teikyo University, Itabashi, Tokyo, Japan; 2 Department of Ophthalmology, School of Medicine, Teikyo University, 2-11-1 Kaga, Itabashi, Tokyo, 173-8605 Japan; 3 Division of Orthoptics, Graduate School of Medical Care and Technology, Teikyo University, Itabashi, Tokyo, Japan

**Keywords:** Preclinical research, Translational research

## Abstract

In the evaluation of smooth pursuit eye movements (SPEMs), recording the stimulus onset time is mandatory. In the laboratory, the stimulus onset time is recorded by electrical signal or programming, and video-oculography (VOG) and the visual stimulus are synchronized. Nevertheless, because the examiner must manually move the fixation target, recording the stimulus onset time is challenging in daily clinical practice. Thus, this study aimed to develop an algorithm for evaluating SPEMs while testing the nine-direction eye movements without recording the stimulus onset time using VOG and deep learning–based object detection (single-shot multibox detector), which can predict the location and types of objects in a single image. The algorithm of peak fitting–based detection correctly classified the directions of target orientation and calculated the latencies and gains within the normal range while testing the nine-direction eye movements in healthy individuals. These findings suggest that the algorithm of peak fitting–based detection has sufficient accuracy for the automatic evaluation of SPEM in clinical settings.

## Introduction

Eye movements include the ability to fixate and track visual stimuli. In most ophthalmology clinics, the examiner evaluates smooth pursuit eye movements (SPEMs) by subjectively noting their accuracy in relation to a target that is being moved manually to nine directions by the examiner. At the same time, the patient follows it with his or her eyes^1–8^. Nevertheless, because the laboratory methods for quantifying eye movements are constrained by the presentation of the target, no objective evaluation method has been established for use in daily clinical practice^9–11^.

To achieve high accuracy in eye movement testing, it is necessary to present a predetermined target on the monitor according to the programming code.Nevertheless, presenting targets according to the prescribed protocol is difficult in the clinical setting, because the examiner must modify the movement of the target as appropriate to examine the suspected abnormality^12–16^.

In the approach to obtaining an accuracy of eye movement measurement in the clinical field that is close to the laboratory level, Hirota et al.^17^ reported that a single-shot multibox detector (SSD)^18^, which is an algorithm for deep learning–based object detection, achieved high accuracy in recognizing the target that was moved manually, and the target location was significantly and highly positively correlated with the positions of both eyes, as recorded by the video-oculography (VOG; the VOG-SSD system). Moreover, the processing speed of SSD exceeds 30 frame per second (fps) and is likely to withstand use in a clinical setting. However, previous studies by Hirota et al. mainly focused on recording the target location and eye movements simultaneously in a clinical setting, with the analysis of eye movements performed manually^17^. The VOG-SSD system should be automated for both measurement and analysis in order to be utilized in clinical practice due to the large amounts of data generated from the VOG and SSD measurements.

Latency and gain are indicators to assess eye movement abnormalities; the stimulus onset time is necessary to evaluate these indicators^19,20^. In the laboratory, the stimulus onset time is recorded by the electrical signal, and the VOG and the visual stimulus are synchronized. Nevertheless, because the examiner must manually move the fixation target, recording the stimulus onset time is challenging in daily clinical practice. Thus, this study aimed to develop an algorithm for evaluating SPEM in nine-gaze direction testing using the VOG-SSD system without recording the stimulus onset time.

## Methods

### General procedures

#### Subjects

A total of 23 young adult volunteers (age [mean ± standard deviation], 21.4 ± 1.4 years] participated in this study. All subjects underwent complete ophthalmologic examinations, including determination of the ocular dominance using the hole-in-the-card test, best-corrected visual acuity at a distance (5.0 m), near the point of convergence, stereoscopic acuity at 40 cm (Titmus Stereotest; Stereo Optical Co., Inc., Chicago, IL, USA), heterophoria by the alternating cover test at near (33 cm) and at distance (5.0 m) assessments, and fundus examinations. Stereoacuity was converted into the logarithm of the arc second (log arcsec).

Table 1 presents the characteristics of the subjects. The mean ± standard deviation of the refractive errors (spherical equivalents) of the dominant eye was − 3.23 ± 3.00 D and that of the nondominant eye was − 3.08 ± 2.80 D. The best-corrected visual acuity was 0.0 logMAR units or better in all subjects. The average heterophoria was − 6.3 ± 5.9 prism diopter (PD) at distance and − 10.9 ± 8.8 PD at near. All healthy volunteers had a stereoacuity of 1.62 ± 0.05 log arcsec (range, 40–60 s).

**Table 1:** Subject characteristics.

ID	Age (years)	SE (D)		Angle of deviation (PD)		Stereoacuity (log arcsec)
		Dominant eye	Nondominant eye	Near	Far	
S1	27	− 0.50	− 0.50	− 4	− 4	1.60
S2	21	− 1.00	− 0.50	− 2	− 4	1.60
S3	23	+ 0.50	+ 0.50	− 4	− 2	1.60
S4	22	− 0.625	− 0.625	− 6	− 14	1.70
S5	20	− 4.375	− 2.875	− 6	− 6	1.60
S6	22	− 0.75	− 0.50	0	0	1.60
S7	21	− 0.125	− 0.875	− 6	− 6	1.60
S8	21	− 6.125	− 5.875	− 8	− 16	1.60
S9	21	− 3.75	− 3.125	− 2	− 4	1.60
S10	21	− 3.375	− 3.375	− 8	− 10	1.60
S11	21	− 7.00	− 7.625	− 6	− 10	1.60
S12	21	− 4.875	− 3.875	− 10	− 18	1.60
S13	21	− 0.375	− 1.125	− 2	− 2	1.60
S14	21	− 1.25	− 1.125	− 8	− 18	1.60
S15	21	− 3.25	− 3.25	− 2	− 8	1.60
S16	21	− 4.50	− 4.75	− 1	− 6	1.70
S17	21	− 5.25	− 4.75	0	− 4	1.70
S18	20	− 3.875	− 3.125	− 14	− 20	1.60
S19	22	− 11.5	− 10.125	− 8	− 18	1.78
S20	20	− 0.125	0.00	− 4	− 10	1.60
S21	21	− 3.00	− 4.125	− 2	− 4	1.60
S22	21	− 0.125	− 0.875	− 6	− 6	1.60
S23	21	− 8.50	− 8.50	− 18	− 25	1.60
Mean	21.3	− 3.23	− 3.09	− 10.9	− 6.3	1.62
SD	1.4	3.00	2.80	8.8	5.9	0.05

Minus and plus signs in the angle of deviation indicate exodeviation and esodeviation of phoria, respectively. A stereoacuity of 1.60, 1.70, and 1.78 log arcsec is equal to 40, 50, and 60 s, respectively.

*S* subject, *SE* spherical equivalent, *D* diopter, *PD* prism diopter, *log arcsec* logarithm of arc second, *SD* standard deviation.

After we explained the nature of the study and possible complications to the subjects, all subjects provided informed consent. This investigation adhered to the World Medical Association Declaration of Helsinki tenets. The Institutional Review Board of Teikyo University approved the experimental protocol and consent procedures (approval No. 19–224–2).

### Apparatus

In this study, we used the VOG-SSD system developed by Hirota et al.^17^ We recorded eye movements while tracking the target using a VOG (EMR-9, NAC Image Technology Inc., Tokyo, Japan). The VOG device determined the eye positions by detecting the corneal reflex and pupil center that were created by the reflection of near-infrared light with a sampling rate of 240 Hz. The measurement error (interquartile range) was 0.2°–0.5° at a distance of 1.0 m. The scene camera recorded the real scenes (resolution, 640 × 480 pixels; angle of view, ± 31° from the center of the scene camera) with a sampling rate of 29.97 Hz. The gaze positions were merged with the real scenes at a delay of ≤ 52 ms.

Before performing the eye movement test, all subjects underwent a calibration test to adjust the position of their gaze on the images of the scene camera and under binocular conditions with fully corrected glasses. All subjects were asked to fixate on nine red cross targets (visual angle, 0.1°) on a white calibration plate at 1.0 m during calibration. From one to nine, the nine red crosses of the targets were set at the following parameters: (center: horizontal of 0.0°, vertical of 0.0°), (left: − 20.0°, 0.0°), (right: 0.0°, 20.0°), (upper left: − 20.0°, 20.0°), (upper right: 20.0°, 20.0°), (lower left: − 20.0°, − 20.0°), (lower right: 20.0°, − 20.0°), (upper: 20.0°, 0.0°), and (lower: 0.0°, − 20.0°) respectively. The center of the calibration plate was defined as 0°, the right and upper halves of the screen were defined as the positive sides, and the left and lower halves were defined as the negative sides.

The object detection algorithm used for the SSD^18^ model is the same as that used in Hirota et al.^17^, which detected the target of the rabbit-like character with an accuracy of 99.7% ± 0.6%. The accuracy of calculating the target location in an ideal environment was *R*^2^ = 0.998 (Supplementary Fig. 1).

We used Python 3.8.5 for Windows 10 (Microsoft, Redmond, WA, USA) with the following libraries: Matplotlib 3.3.2, Numpy 1.18.5, OpenCV 3.3.1, Pandas 1.1.3, Pytorch 1.6.0, Scikit-learn 0.23.2, and Seaborn 0.11.0.

### Nine-direction eye movements testing

The target was a rabbit-like character that had already been learned to the SSD in Hirota et al.^17^ The target size was 10 × 10 cm, which subtended a visual angle of 5.7° at 1.0 m. The target was manually moved to nine directions (center, left, right, upper left, upper right, lower left, lower right, upper, and lower) within ± 15° randomly by an examiner.

All subjects were seated in a well-lit room (600 lx) wearing fully corrective spectacles. Each subject's head was stabilized with a chin rest and forehead rest. During the eye movement test, the subjects were asked to fixate on the nose of the target, the visual angle of which was 0.1° at 1.0 m.

### Filtering for both eye positions

We excluded VOG data when the change in pupil diameter was > 2 mm/frame due to blinking^21^. We replaced the percentage of missing values (0.4% ± 0.7% for all subjects) with a linearly interpolated value calculated from an algorithm written with Python 3.8.5. The horizontal and vertical eye movements were analyzed, and the SPEM and saccadic eye movements were identified using a velocity-threshold identification (I-VT) filter^22^. The I-VT filter was used to classify eye movements on the basis of the velocity of the directional shifts of the eye. A saccadic eye movement was defined as the median velocity of three consecutive windows > 100°/s. Then, the eye position data at 240 Hz were synchronized with the target data at 29.97 Hz.

## Experiment 1

Eye movement testing involves moving the target in eight directions: left, right, upper left, upper right, lower left, lower right, upper, and lower. There is a need for an algorithm that can identify the direction in which the examiner moves the target manually in the clinic without the input of a trigger. In experiment 1, we compared the accuracy of the classification in each direction of target presentation between the peak fitting–based detection algorithm and the conventional threshold-based detection algorithm.

### Procedures

**Figure 1:**
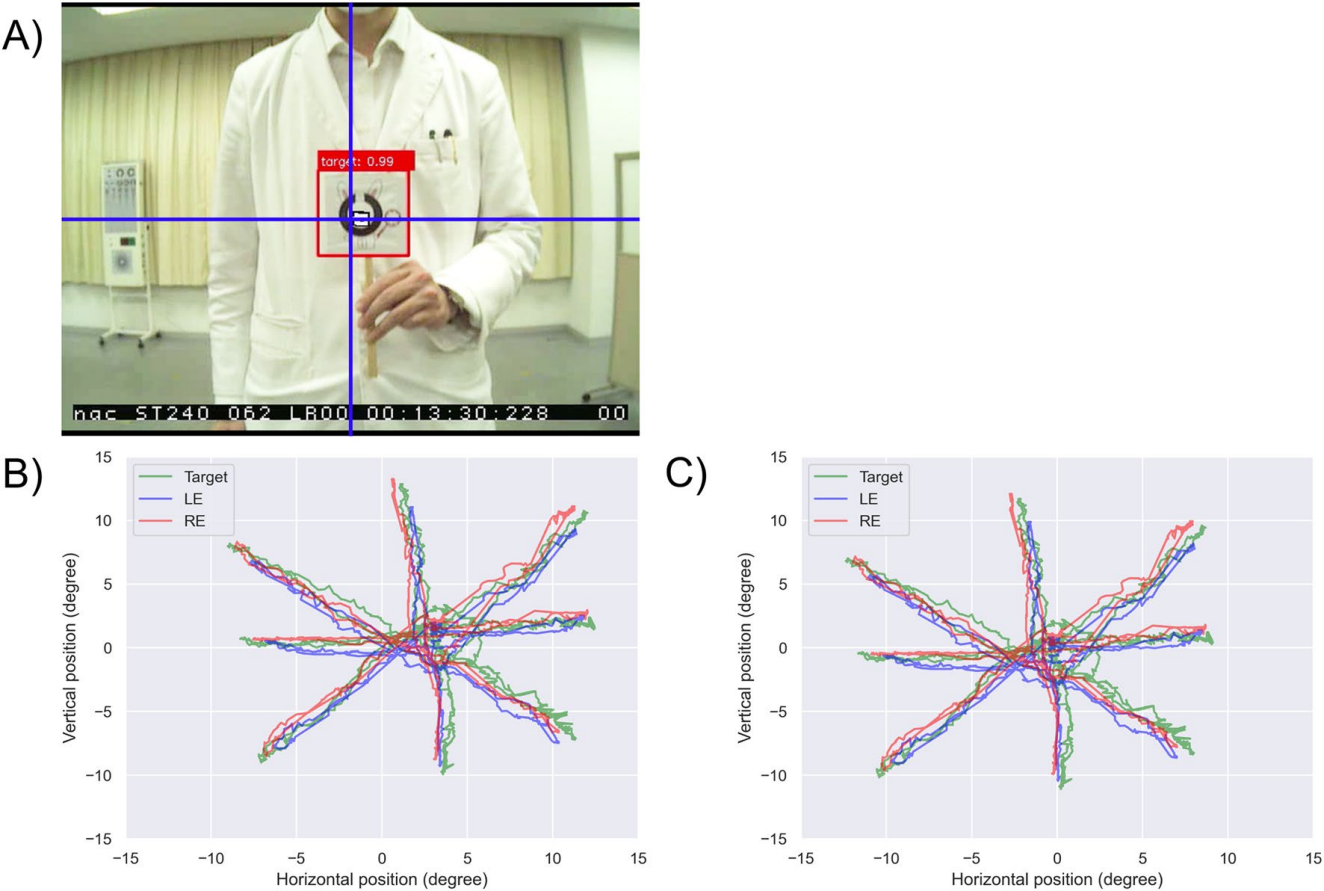
Setting the relative origin. In clinical settings, the target that is presented by the examiner (**A**) does not always coincide with the center of the scene camera (**B**). The median of the target location was calculated both horizontally and vertically, respectively, and was defined as the relative origin (**C**).

In clinical practice, the origin of the scene camera (horizontal of 0.0°, vertical of 0.0°) and the position where the target is initially presented by the examiner do not necessarily coincide (Fig. 1A, B). The median of the target location of the target was calculated both horizontally and vertically, respectively, and was defined as the relative origin. The target location and both eye positions were corrected for the difference from the relative origin (Fig. 1C).

The target location calculated using the SSD was identified more than 99% of the time and was more stable than eye positions affected by blinks and tears. Thus, each direction was identified using the location of the target as a cue.

### Algorithm of automatic detection for testing the directions of eye movements

#### Peak fitting–based detection

**Figure 2:**
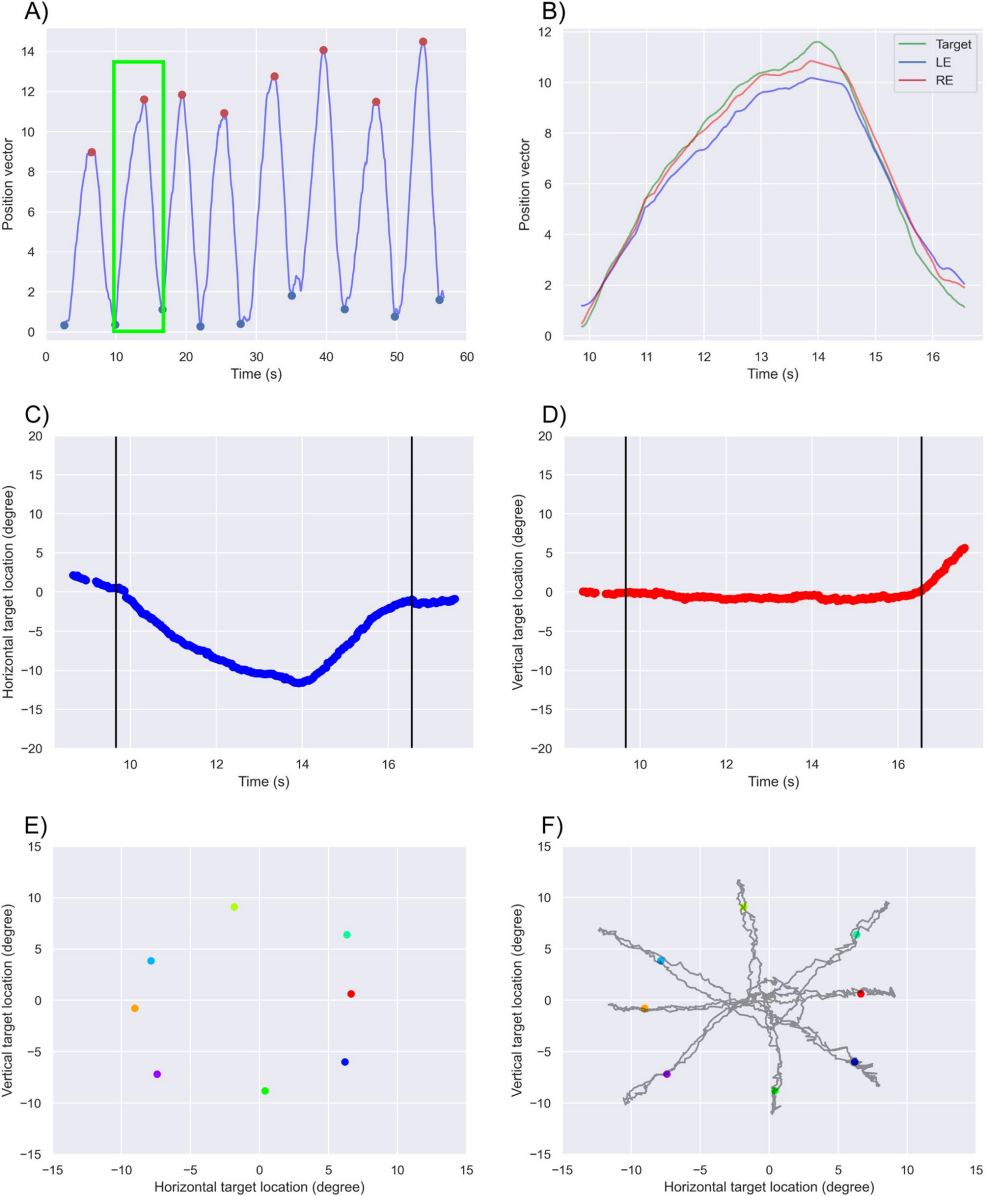
Algorithm of peak fitting–based detection. The target location was converted to the position vector, and then, the maximum and minimum peaks were detected for 3.0 s (**A**). (**B**) Data between two minimum peaks, including one maximum peak in the green square of (**A**). The separated data were decomposed into horizontal (**C**) and vertical (**D**) components from the position vector. After excluding 1 s from both ends of the separated data (black vertical lines in **C** and **D**), the medians of the horizontal and vertical target locations were calculated and plotted (**E**). (**F**) Superimposition of the relative origin data, which is the same as Fig. 1C of the target.

The target location was converted to the position vector, and then, the maximum and minimum peaks were detected for 3.0 s (Fig. 2A, B). We separated the data between the two minimum peaks, including one maximum peak. The separated data were decomposed into horizontal and vertical components from the position vector (Fig. 2C, D). After excluding 1 s from both ends of the separated data, the medians of the horizontal and vertical target locations were calculated (Fig. 2E, F).

**Figure 3:**
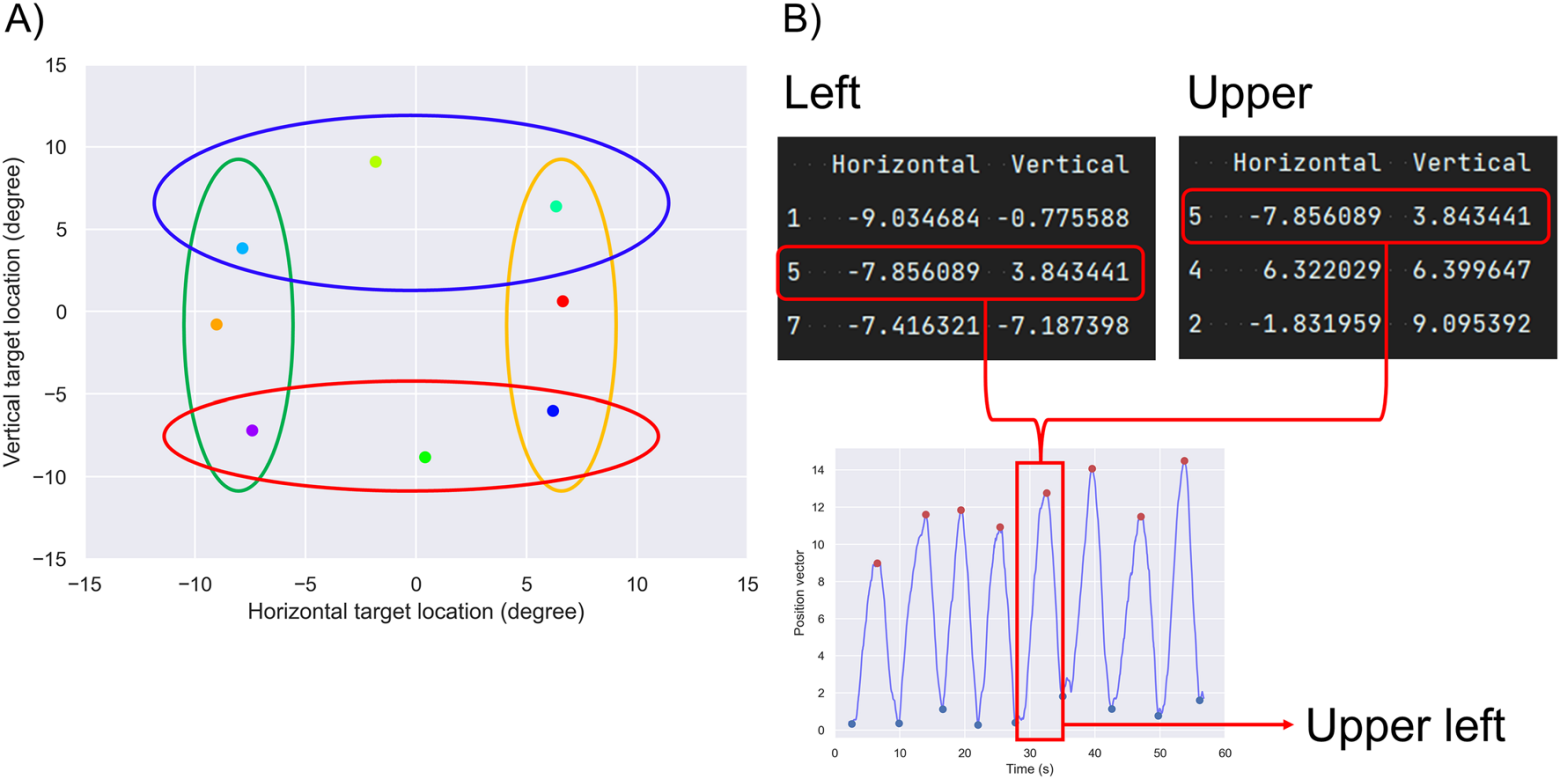
Categorizing each direction. The eight horizontal and vertical median locations were ranked from maximum to minimum at left (green ellipse), right (yellow ellipse), upper (blue ellipse), and lower (red ellipse), and then, the top three values in the four directions were grouped (**A**). Then, the plots that belong to the two groups (upper left, upper right, lower left, and lower right) were identified by combining the horizontal and vertical directions (the red squares in **B**). Each plot was numbered according to the order of the maximum peak calculated in Fig. 2A; thus, the fifth waveform is in the upper left.

The eight median horizontal and vertical locations were ranked from maximum to minimum at left, right, upper, and lower, and then the top three values in four directions were grouped (Fig. 3A). The upper left, upper right, lower left, and lower right were identified by combining the horizontal and vertical directions (Fig. 3B). The remaining data in each group were the left, right, upper, and lower.

#### Threshold-based detection

Threshold-based detection is a simple approach for identifying the category. In this study, the target data were separated to left (horizontal location ≤  − 2.0° and − 2.0° ≤ vertical location ≤  + 2.0°), right (+ 2.0 ≤ horizontal location and − 2.0° ≤ vertical location ≤  + 2.0°), upper left (horizontal location ≤  − 2.0° and + 2.0° ≤ vertical location), upper right (+ 2.0 ≤ horizontal location and + 2.0° ≤ vertical location), lower left (horizontal location ≤  − 2.0° and vertical location ≤  − 2.0°), lower right (+ 2.0 ≤ horizontal location and vertical location ≤  − 2.0°), upper (− 2.0 ≤ horizontal location ≤  + 2.0° and + 2.0° ≤ vertical location), and lower (− 2.0 ≤ horizontal location ≤  + 2.0° and vertical location ≤  − 2.0°). The cutoff value was defined as the minimum value calculated using the averaged degree of the mean − 2.0 standard deviation in all directions using the data from subject 1 to subject 5.

### Statistical analysis

In this study, classification accuracy was assessed for 23 subjects in eight directions (for a total 184 directions), excluding the center. The classification results of each algorithm were divided into correct and incorrect answers using the direction in which the target was actually presented as the ground truth. We evaluated the accuracy of the classification in each direction between the peak fitting–based and threshold-based detection using Fisher's exact test with the degree of feedom was set 1.

SPSS version 26 (IBM Corp., Armonk, NY, USA) was used to determine the significance of the differences, and a *P* value of < 0.05 was considered to be statistically significant.

### Results

The accuracy of the classification in each direction was significantly higher using the peak fitting–based detection (correct, 100.0%; incorrect, 0.0%) than using the threshold-based detection (correct, 47.8%; incorrect, 52.2%; *P* < 0.001, Fisher’s exact test; Table 2).

**Table 2:** Accuracy of the classification for each direction in all subjects.

Algorithm	Correct	Incorrect
Peak	184	0
Threshold	88	96

A total of 23 healthy subjects participated in this study. The algorithms identified eight directions in each subject. Hence, 184 data points were analyzed. The algorithm of peak fitting–based detection was classified correctly in all directions of target orientation.

*peak* peak fitting–based detection, *threshold* threshold-based detection.

The finding of experiment 1 suggested that the algorithm of the peak fitting–based detection was suitable for evaluating eye movement testing.

## Experiment 2

In experiment 2, we investigated the algorithm for the automatic calculation of latency and gain, which are evaluation indices of the eye movements using the data obtained by the peak fitting–based detection algorithm.

### Calculating for latency and gain

**Figure 4:**
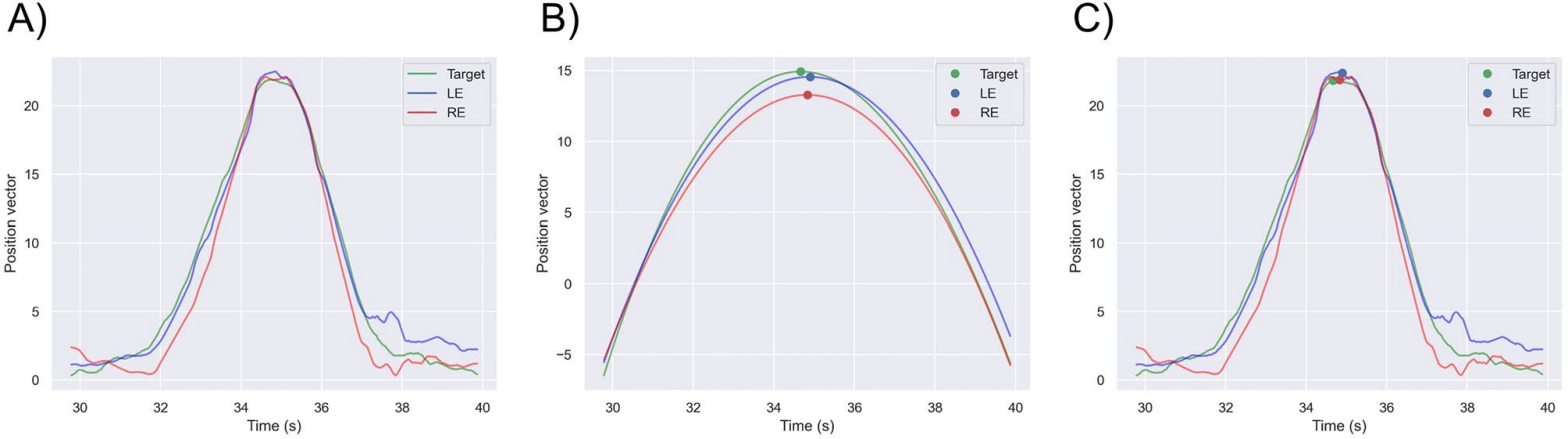
Calculating latency. The horizontal and vertical target locations and both eye positions were converted to the position vector (**A**). (**A**) were fitted with a cubic function and detected at each peak time (**B**). (**C**) Each peak time was applied to (**A**). The latencies of both eyes were defined as the difference between the peak time in both eyes and that in the target location.

All directions of the horizontal and vertical target location and both eye positions were converted to the position vector. The raw data were fitted with a cubic function and were detected at each peak time (Fig. 4A, B). Then, each peak time was applied to the raw data (Fig. 4C). The latencies of both eyes were defined as the difference between the peak time in both eyes and that in the target location.

**Figure 5:**
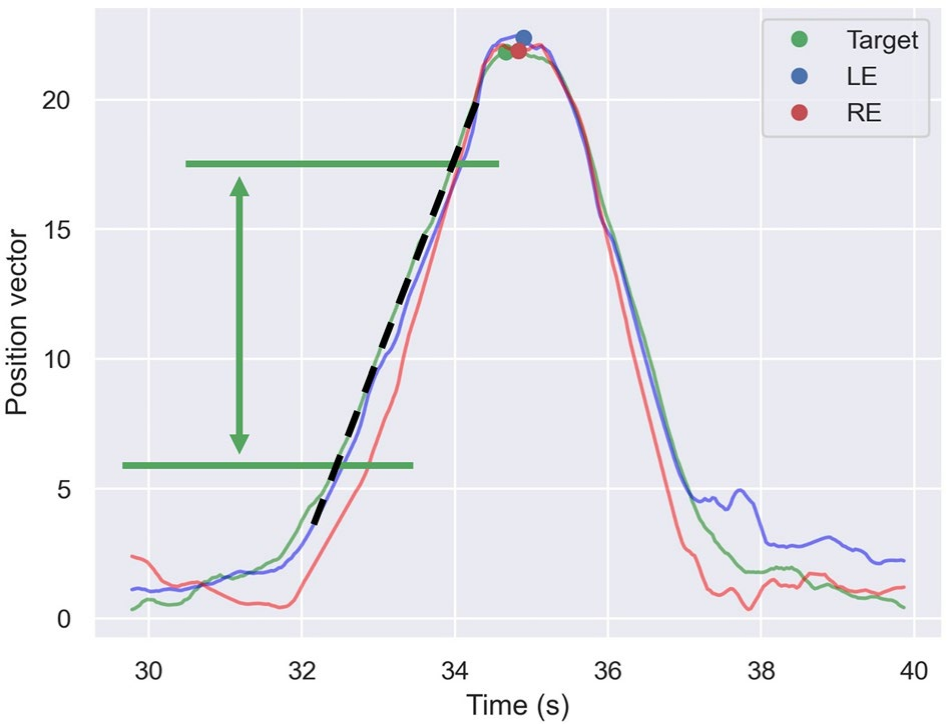
Calculating gain. The target location and both eye positions at the peak time were defined as maximum values. The 25th and 75th percentile points of the maximum values in the centrifugal direction were explored (the green horizontal lines). The gains of both eyes were defined as the ratio of the slope of the regression line in both eyes to the slope of the regression line in the target (the black dashed line) between the 25th and 75th percentile points.

The target location and both eye positions at the peak time were defined as maximum values. We explored the 25th and 75th percentile points of the maximum values in the centrifugal direction (Fig. 5). We then created a linear regression line using the target location and both eye positions between the 25th and 75th percentile points of the maximum values. The gains of both eyes were defined as the ratio of the slope of the regression line in both eyes to the slope of the regression line in the target between the 25th and 75th percentile points.

### Statistical analysis

We determined the differences in the latencies and gains within both eyes in each direction using the Schéffe test. We analyzed the differences in the latencies and gains between both eyes in each direction using the Wilcoxon signed-rank test with Bonferroni correction used to adjust the *P* values.

### Results

**Figure 6:**
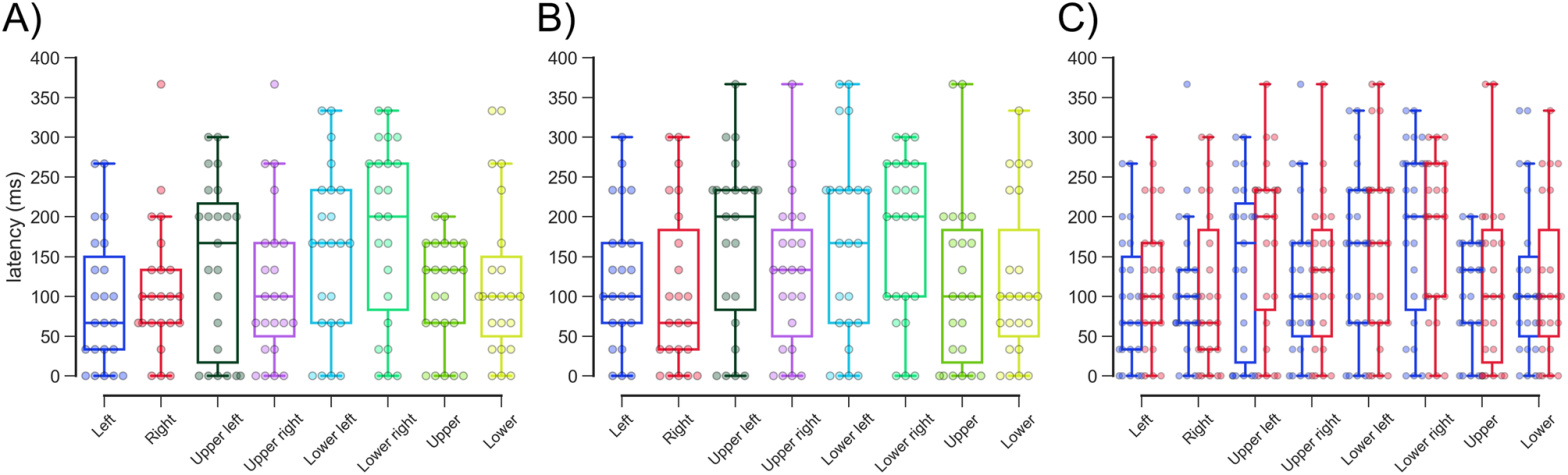
Latencies of the left (**A**) and right (**B**) eyes in each direction. The latencies in all directions were not significantly different within both eyes (**A** and **B**). The blue and red boxplots with dots indicate the latency of the left and right eyes in each direction (**C**). The latencies in all directions were not significantly different between the left and right eyes.

The latencies in all directions were not significantly different within both eyes (left eye, *P* > 0.22; right eye, *P* > 0.70; Schéffe test; Fig. 6A, B; Table 3). The latencies in all directions were not significantly different between left (127.17 ± 98.53 ms in all directions) and right (137.14 ± 104.80 ms in all directions) eyes (*P* > 0.21, Wilcoxon signed-rank test with Bonferroni correction; Fig. 6C; Table 3).

**Table 3:** Latencies in all directions.

Direction	Latency (ms)		*P* value
	LE	RE	
Left	94.20 ± 82.62	123.19 ± 85.37	0.21
Right	108.70 ± 82.37	107.25 ± 99.74	> 0.99
Upper left	140.58 ± 105.39	169.56 ± 107.64	> 0.99
Upper right	113.04 ± 95.67	127.54 ± 92.55	> 0.99
Lower left	155.07 ± 103.38	162.32 ± 119.70	> 0.99
Lower right	182.61 ± 112.02	173.91 ± 100.22	> 0.99
Upper	102.90 ± 66.60	115.94 ± 106.72	> 0.99
Lower	120.29 ± 99.15	117.39 ± 98.23	> 0.99

The error term is the standard deviation. *P*-values were calclated using Wilcoxon signed rank test with Bonferroni correction.

*ms* milliseconds, *LE* left eye, *RE* right eye.

**Figure 7:**
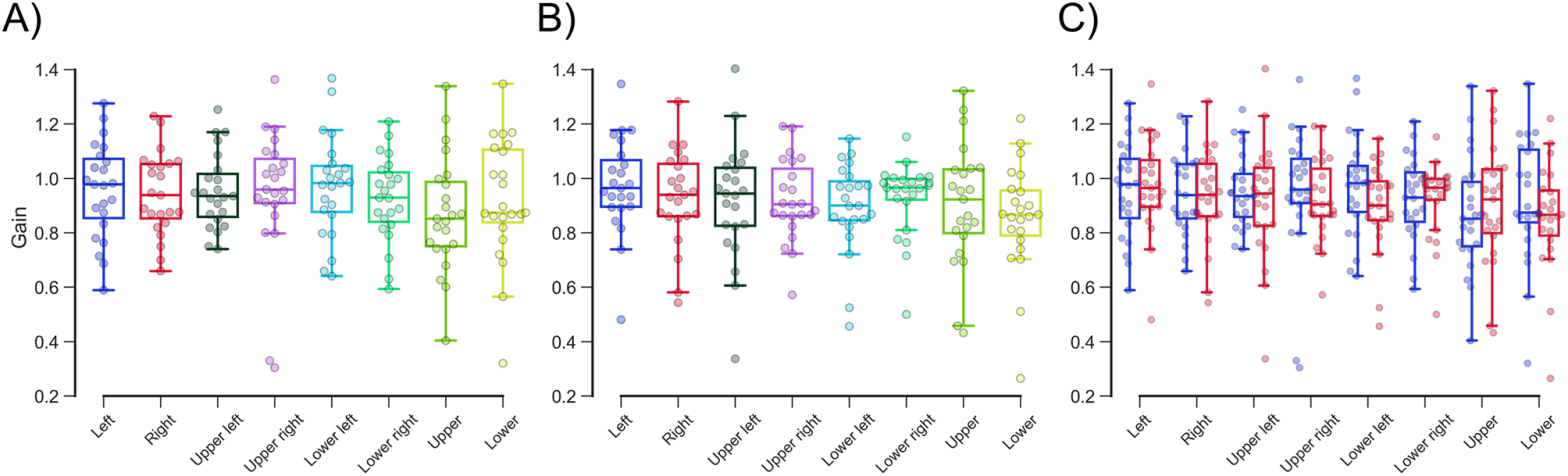
Gains of the left (**A**) and right (**B**) eyes in each direction. The gains in all directions were not significantly different within both eyes (**A** and **B**). The blue and red boxplots with dots indicate the latency of the left and right eyes in each direction (**C**). The gains in all directions were not significantly different between left and right eyes.

The gains in all directions were not significantly different within both eyes (left eye, *P* > 0.85; right eye; *P* > 0.68, Schéffe test; Fig. 7A, B; Table 3). The gains in all directions were not significantly different between left (0.936 ± 0.186 in all directions) and right (0.916 ± 0.180 in all directions) eyes (*P* > 0.52, Wilcoxon signed-rank test with Bonferroni correction; Fig. 7C; Table 4).

**Table 4:** Gains in all directions.

Direction	Gain		*P* value
	LE	RE	
Left	0.957 ± 0.171	0.975 ± 0.171	> 0.99
Right	0.942 ± 0.148	0.931 ± 0.169	> 0.99
Upper left	0.953 ± 0.135	0.918 ± 0.212	> 0.99
Upper right	0.949 ± 0.233	0.921 ± 0.146	0.76
Lower left	0.971 ± 0.182	0.895 ± 0.162	0.52
Lower right	0.925 ± 0.153	0.929 ± 0.113	> 0.99
Upper	0.869 ± 0.205	0.904 ± 0.217	> 0.99
Lower	0.920 ± 0.221	0.857 ± 0.196	> 0.99

The error term is the standard deviation. *P*-values were calclated using Wilcoxon signed rank test with Bonferroni correction.

*ms* milliseconds, *LE* left eye, *RE* right eye.

The findings of experiment 2 suggest that using the algorithm of peak fitting–based detection, the eye movements can be evaluated from the data with the identified target direction.

## Additional experiment

One patient with postsurgical congenital superior oblique muscle palsy underwent an additional experiment to investigate the scope of clinical applicability. The patient underwent a complete ophthalmologic examination, including determination of ocular dominance using the hole-in-the-card test, best-corrected visual acuity at a distance, the near point of convergence, stereoscopic acuity at 40 cm, heterotropia using the alternate cover test near and at a distance, and fundus examination.

The dominant eye was the right eye, as the left eye had undergone surgery for strabismus 30 years ago. The patient was examined with a natural head position so that binocular vision could be maintained. Since the patient had abnormal head positions, the following positions were used: face turned to the right, head tilted to the right, and chin down. The spherical equivalent of the dominant eye was − 0.75 D and that of the nondominant eye was − 0.75 D. The best-corrected visual acuity was 0.0 logMAR in each eye. The horizontal and vertical heterotropia measures were 1.0 PD base-out and 1.0 PD base-up at distance and 4.0 PD base-in and 7.0 PD base-up near. Stereo acuity was 1.60 log arcsec (40 s).

**Figure 8:**
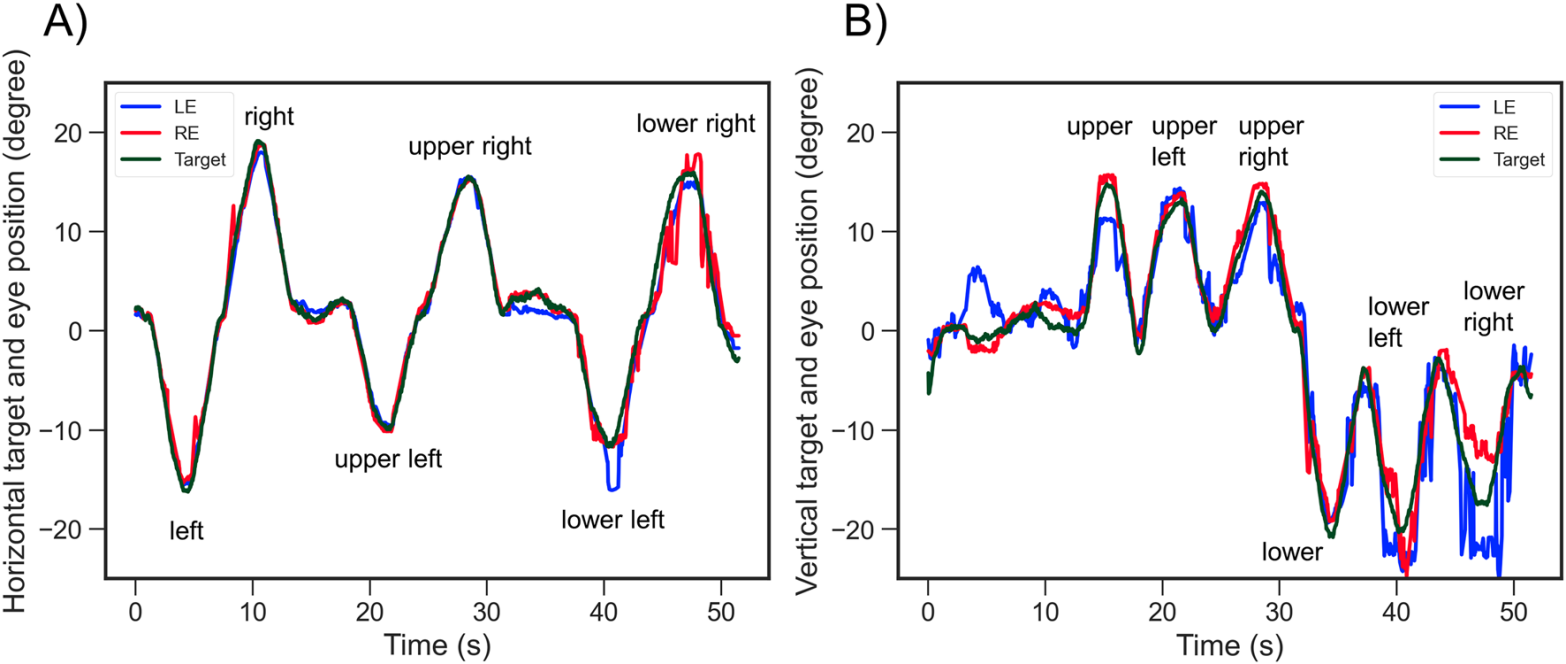
Horizontal (**A**) and vertical (**B**) eye movements in the patient with postsurgical congenital superior oblique muscle palsy. The green, blue, and red lines indicate the target location, left eye position, and right eye position, respectively. The left eye had previously undergone strabismological surgery.

Figure 8 shows the patient’s horizontal and vertical eye movements. The latencies in the lower left (533.33 ms), lower right (633.33 ms), and lower (233.33 ms) areas of the nondominant eye were prolonged in comparison to those of the dominant eye (lower left, 266.67 ms; lower right, 66.67 ms; lower, 66.67 ms). The gains in nondominant eye of lower left (533.33 ms), lower right (633.33 ms), and lower (233.33 ms) were prolonging comparison to dominant eye (lower left, 266.67 ms; lower right, 66.67 ms; lower, 66.67 ms) (Table 5). In addition, the gains in the lower right and lower areas of the nondominant eye were slower than those in the dominant eye (Table 5). The gains between the nondominant and dominant eye did not show a clear trend (Table 5).

**Table 5:** Latencies and gains in the patient with postsurgical congenital superior oblique muscle palsy.

Direction	Latenct (ms)		Gain	
	LE	RE	LE	RE
Left	66.67	66.67	0.948	0.843
Right	0.00	0.00	0.929	0.281
Upper left	66.67	66.67	1.014	0.760
Upper right	66.67	100.00	1.034	0.792
Lower left	533.33	266.67	0.887	0.185
Lower right	633.33	66.67	0.667	0.649
Upper	100.00	100.00	0.927	0.821
Lower	233.33	66.67	0.416	0.862

The error term is the standard deviation.

*ms* milliseconds, *LE* left eye, *RE* right eye.

## Discussion

Recording the stimulus onset time is mandatory when evaluating eye movements. Nevertheless, because the examiner must move the fixation target manually, recording the stimulus onset time is challenging in daily clinical practice. In this study, we developed an algorithm of peak fitting–based detection to evaluate SPEM in nine-direction testing without recording the stimulus onset time. We found that the present algorithm had high accuracy for identifying the directions of target orientation in nine-direction testing.

The classification accuracy of the threshold–based detection algorithm was about half that of the peak fitting–based detection algorithm (Table 2). There are no standard criteria for determining the threshold; however, in this study, the threshold was determined from the standard deviation of five subjects. Since the standard deviation is based on 68% of the total data, the decrease in the classification accuracy of the threshold-based detection algorithm to the chance level indicates the significant influence of the oblique direction in the automatic determination of nine-direction testing.

The peak fitting–based detection algorithm correctly classified all directions of the target orientation (Table 2). This finding suggests that the peak fitting–based detection algorithm shows superiority for determining the oblique direction: to identify the oblique direction, listing the top three waveforms of the left, right, upper and lower, and then the waveforms existing in the combinations left and upper, left and lower, right and upper, and right and lower are the upper left, lower left, upper right, and lower right, respectively (Fig. 3).

The latencies (mean latencies of left and right eyes, 138.04 and 144.75 ms, respectively) and gains (mean gains of left and right eyes, 0.943 and 0.935, respectively), which we calculated from the data of the identified target direction using the algorithm of peak fitting–based detection, were similar to those reported in earlier studies: the latency of the SPEM was between 50 and 300 ms^19,23,24^, the gain of SPEM was greater than 0.90 under a velocity of 10°/s, and the moving distance was 15° in healthy individuals^20^. These results suggest that the accuracy of the automatic method for calculating latency and gain is consistent with that of the manual analysis method.

We evaluated only one patient with postsurgical congenital superior oblique muscle palsy. The latencies in nondominant eye of lower left, lower right, and lower were prolonged comparison to dominant eye (Table 5). The eye movements in nondominant eye that had been undergone strabismological surgery were unstable when looking downward (Fig. 8). These findings may suggest that evan if the patient is obtained binocular vision in the primary eye position by stabismological surgery, oculomotor deficits in the working direction of the paralytic muscles remain. On the other hand, the gains in patient did not show a clear trend. The earlier studies reported that the paralystic strabismus mixes with saccade in SPEM^25,26^. We plan to investigate the characteristics of eye movements in paralytic strabismus.

The peak fitting–based detection algorithm classified all directions of the target orientation and calculated the latency and gain in a way similar to the manual analysis in healthy individuals. However, there is concern that, depending on the degree of ocular motility disorder, the peak of the waveform for calculating latency and gain may not be detected. Thus, in a future study, we plan to investigate the accuracy of automatic analysis in patients with ocular motility disorders.

## Conclusion

The algorithm of peak fitting–based detection correctly classified the directions of the target orientation and calculated the latencies and gains within the normal range during nine-direction eye movement testing in healthy individuals. These findings suggest that the peak fitting–based detection algorithm has an accuracy that is sufficient for the automatic evaluation of SPEM in clinical settings.

## Supplementary Information

Supplementary Information 1.

Supplementary Information 2.

## Data Availability

The data that support the findings of this study are openly available in Zenodo (https://doi.org/10.5281/zenodo.6400751).
