# Supplementary material for: Analysis of smooth pursuit eye movements in a clinical context by tracking the target and eyes
Source: Sci Rep. 2022 May 19;12:8501. doi: 10.1038/s41598-022-12630-6 (PMC9120200; doi:10.1038/s41598-022-12630-6)
Supplement: Supplementary file 1 — Supplementary Information 1. [file 41598_2022_12630_MOESM1_ESM.docx]

**Supplementary Figure Legends**

**Supplementary Figure 1. Correlation between calculated horizontal (A) and vertical (B) target locations using SSD and actual target locations.**

We compared the ideal target location and the target location using SSD. The target was a rabbit-like character. The target size was 10 × 10 cm, which subtended a visual angle of 5.7 degrees at 1.0 m. The target was displayed on a 24-inch liquid crystal monitor. The center of the monitor was defined as 0 degree; the right and upper halves of the monitor were defined as the positive sides; and the left and lower halves were defined as the negative sides. The target was moved ±15 degrees with a random velocity of ≤10 degrees/s, which was preset by a computer. The calculated horizontal (*R^2^* = 0.998, *P* < 0.001; simple linear regression analysis) and vertical (*R^2^* = 0.998, *P* < 0.001; simple linear regression analysis) target locations using SSD was significantly and positively correlated with the true target locations.

SSD, single shot multibox detector.
