# Supplementary figures and images for: Analysis of smooth pursuit eye movements in a clinical context by tracking the target and eyes
Source: Sci Rep. 2022 May 19;12:8501. doi: 10.1038/s41598-022-12630-6 (PMC9120200; doi:10.1038/s41598-022-12630-6)

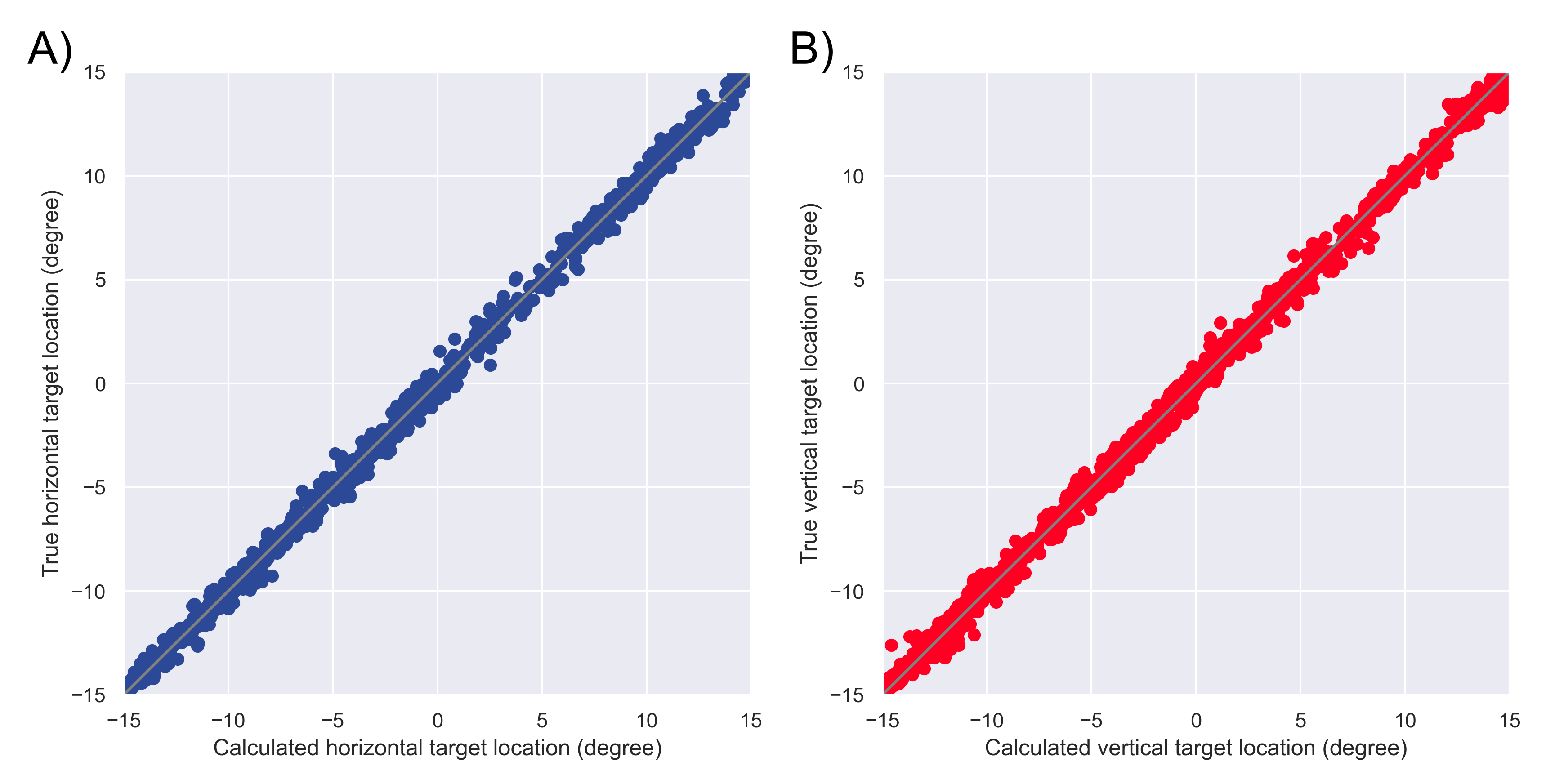

Supplement: Supplementary file 2 — Supplementary Information 2. [file 41598_2022_12630_MOESM2_ESM.tif]
